# Pro-opiomelanocortin and ACTH–cortisol dissociation during pediatric cardiac surgery

**DOI:** 10.1530/EC-24-0078

**Published:** 2024-05-13

**Authors:** Arno Téblick, Ilse Vanhorebeek, Inge Derese, An Jacobs, Renata Haghedooren, Sofie Maebe, Gerdien A Zeilmaker-Roest, Enno D Wildschut, Lies Langouche, Greet Van den Berghe

**Affiliations:** 1 Clinical Division and Laboratory of Intensive Care Medicine, Department of Cellular and Molecular Medicine, KU Leuven, Leuven, Belgium; 2 Department of Neonatal & Pediatric Intensive Care, Division of Pediatric Intensive Care, Erasmus MC – Sophia Children’s Hospital, Rotterdam, the Netherlands

**Keywords:** adrenocorticotropic hormone, cortisol, pediatric cardiac surgery, pediatric critical illness, pro-opiomelanocortin

## Abstract

In critically ill adults, high plasma cortisol in the face of low ACTH coincides with high pro-opiomelanocortin (POMC) levels. Glucocorticoids further lower ACTH without affecting POMC. We hypothesized that in pediatric cardiac surgery-induced critical illness, plasma POMC is elevated, plasma ACTH transiently rises intraoperatively but becomes suppressed post-operatively, and glucocorticoid administration amplifies this phenotype. From 53 patients (0–36 months), plasma was obtained pre-operatively, intraoperatively, and on post-operative days 1 and 2. Plasma was also collected from 24 healthy children. In patients, POMC was supra-normal pre-operatively (*P* < 0.0001) but no longer thereafter (*P* > 0.05). ACTH was never high in patients. While in glucocorticoid-naive patients ACTH became suppressed by post-operative day 1 (*P* < 0.0001), glucocorticoid-treated patients had already suppressed ACTH intraoperatively (*P* ≤ 0.0001). Pre-operatively high POMC, not accompanied by increased plasma ACTH, suggests a centrally activated HPA axis with reduced pituitary processing of POMC into ACTH. Increasing systemic glucocorticoid availability with glucocorticoid treatment accelerated the suppression of plasma ACTH.

**Significance statement:**

Glucocorticoids are often administered during pediatric cardiac surgery. In critically ill children, endogenous systemic glucocorticoid availability is elevated already upon ICU admission while ACTH levels are normal. This hormonal constellation suggests the presence of active feedback inhibition of ACTH. In this study, we have documented that intraoperative administration of glucocorticoids accelerates the suppression of ACTH, resulting in low plasma ACTH already upon ICU admission. Pre-operative plasma POMC, the ACTH precursor, but not ACTH, was increased. This is compatible with a centrally activated HPA axis prior to surgery in young children but reduced processing of POMC into ACTH within the pituitary. These findings suggest that glucocorticoid treatment in the context of pediatric cardiac surgery may amplify pre-existing impaired pituitary processing of the prohormone POMC.

## Introduction

Recently, we have found that during critical illness, the well-known constellation of ‘low plasma ACTH in the face of increased systemic glucocorticoid availability’ coincides with a substantial increase in plasma concentrations of the ACTH precursor, pro-opiomelanocortin (POMC) (1). Results from our mechanistic animal study suggested that during sepsis-induced critical illness, preserved hypothalamic corticotropin-releasing hormone (CRH) and arginine vasopressin (AVP) expression continuously drives pituitary production of POMC, while high circulating levels of free corticosterone suppress the processing of POMC into ACTH and secretion of mature ACTH into the circulation. Concurrent central stimulation of the precursor while processing into the mature product is impaired explains the high circulating levels of the precursor, POMC, and low circulating levels of the mature product, ACTH, during critical illness. These findings were corroborated in two human adult patient studies of acute (daily sampling throughout the first week of the intensive care unit (ICU) stay) and prolonged critical illness (weekly sampling beyond the first week of ICU stay), in which plasma concentrations of POMC were always substantially elevated (1). It remained unclear, however, how early plasma POMC levels start to rise and how this relates to the decline in plasma ACTH levels.

In a subsequent study in sepsis-induced critically ill mice, we demonstrated that early continuous infusion of stress doses of hydrocortisone (1.2 mg/day, ~200–300 mg/day human equivalent (2)) resulted in further suppression of plasma ACTH concentrations after 7 days of critical illness as compared with infusion of placebo (3), without affecting plasma POMC concentrations. These data suggested that further augmentation of systemic glucocorticoid availability by exogenous administration amplifies the impaired pituitary processing of POMC into ACTH. However, whether glucocorticoid treatment in critically ill humans evokes a similar response, i.e. further suppressing plasma ACTH without affecting the elevation in plasma POMC, remained to be studied.

In critically ill children, plasma ACTH is normal upon pediatric intensive care unit (PICU) admission and low thereafter, while free cortisol is only elevated upon PICU admission and normal thereafter (4). Plasma POMC has not been quantified in critically ill children. In addition, the dynamics of plasma ACTH and free cortisol before PICU admission are unknown. In this study performed in children undergoing cardiac surgery, we tested the hypothesis that the critical insult leads to a rise in plasma POMC and a transient intraoperative rise in plasma ACTH followed by post-operative suppression. We further hypothesized that intraoperative/post-operative glucocorticoid administration amplifies this phenotype.

## Methods

### Patients and sample collection

This study was a preplanned secondary analysis of the multicenter Pediatric Analgesia after Cardiac Surgery or ‘PACS’ randomized controlled trial (RCT) conducted in the PICUs of Leuven (Belgium), Rotterdam, Groningen, and Utrecht (the Netherlands). The PACS trial investigated the potential opioid-sparing effect of intermittent i.v. paracetamol administration in children after cardiac surgery. Patients were randomized to receive either i.v. morphine or i.v. paracetamol following cardiac surgery for up to 48 h. Rescue morphine was allowed in both arms. The primary endpoint was the cumulative dose of morphine administered within the first 48 h after surgery.

**Figure 1:**
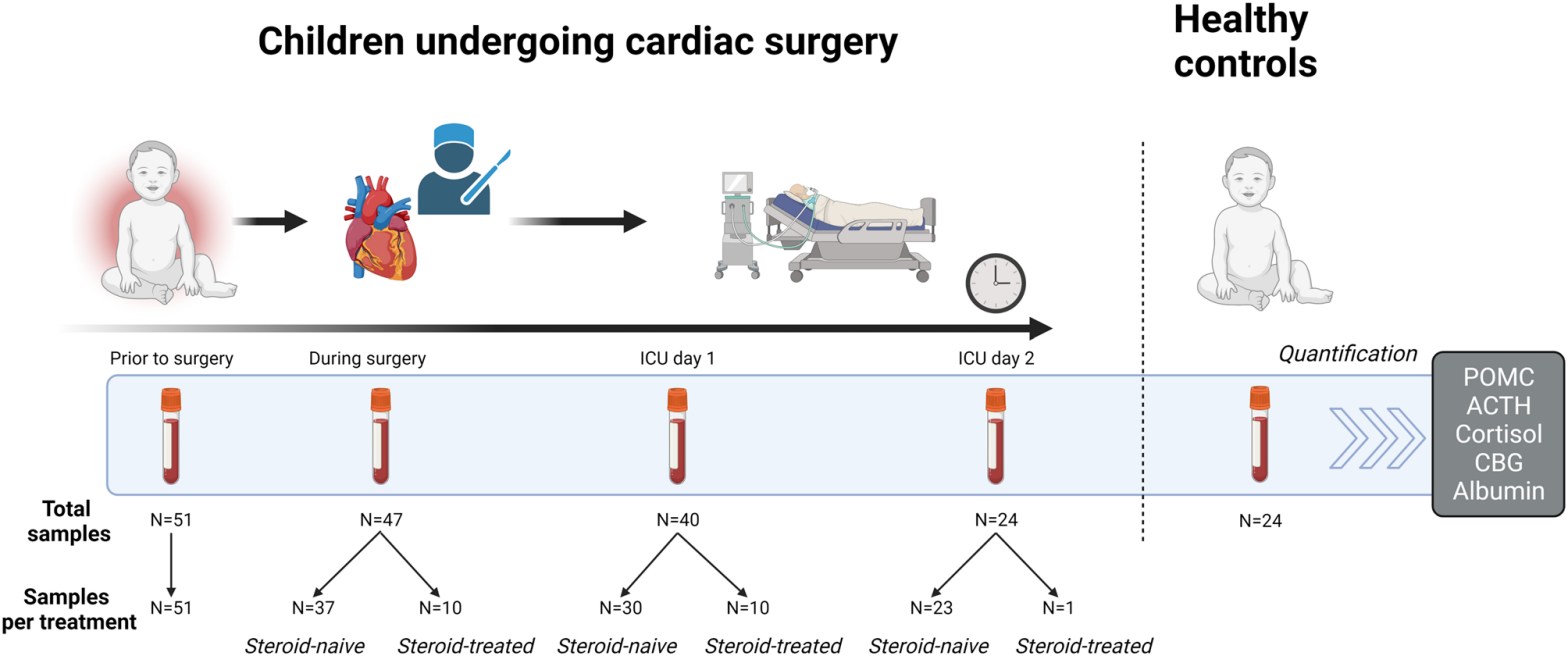
Study design and sample availability.

To document the time profile of POMC, in relation to ACTH and free cortisol, blood samples were obtained in Leuven and Rotterdam from 53 children, aged 0–36 months, who underwent cardiac surgery with cardiopulmonary bypass for any congenital cardiac diagnosis, before, during, and after the surgery on post-operative PICU days 1 and 2 (Fig. 1). Except for plasma POMC on post-operative PICU day 2 (*P* = 0.02) with higher concentrations within the morphine arm, there was no effect of randomization (i.v. morphine vs i.v. paracetamol) on plasma POMC, ACTH, or free cortisol at any time point (all *P* > 0.05). As such, the results of both randomization arms were pooled for the current study. For each time point, patients were further categorized into glucocorticoid-treated or glucocorticoid-naive patients based on whether they received glucocorticoids, which were always synthetic glucocorticoids, within 24 h before sampling (Fig. 1). Administration of glucocorticoids was at the discretion of the anesthesiologist (during the operative phase) or intensive care physician (for PICU days 1 and 2). Blood samples were also obtained from 24 age- and sex-matched healthy children, selected from the PEPaNIC trial, an RCT performed in the same centers (5). Characteristics of patients and healthy children are shown in Table 1.

**Table 1:** Baseline characteristics.

	Healthy controls	Critically ill	*P*
Age (years), median (IQR)	0.64 (0.43–0.84)	0.38 (0.25–0.85)	0.08
Male sex, *n* (%)	13 (54.2%)	50 (50.0%)	0.74
Center, *n* (%)			0.72
Rotterdam	11 (45.8%)	22 (41.5%)	
Leuven	13 (54.2%)	31 (58.5%)	
Primary diagnosis, *n* (%)			
ASD		4 (7.5%)	
AVSD		7 (13.2%)	
VSD		11 (20.8%)	
TOF		13 (24.5%)	
Valvular stenosis/insufficiency		4 (7.5%)	
Other		14 (26.5%)	
RACHS score, *n* (%)			
1		4 (7.7%)	
2		39 (75.0%)	
3		6 (11.5%)	
4		3 (5.8%)	
PIM2 score, median (IQR)		−3.8 (−4.08 to −3.44)	
PIM2 predicted probability of death, %, median (IQR)		2.18 (1.65–3.09)	
Duration (days) of PICU stay, median (IQR)		5 (3–6)	
Duration (days) of hospital stay, median (IQR)		8 (6–14)	

ASD, atrial septal defect, AVSD, atrioventricular septal defect; PIM2, pediatric index of mortality 2; RACHS, risk adjustment for congenital heart surgery; TOF, tetralogy of fallot; VSD, ventricular septal defect.

Written informed consent was obtained from parents or legal guardians. The Institutional Ethical Review Board at each participating center approved the protocol and consent forms of the study (S60861 and NL 53085.078.15, ML8052 and NL38772.000.12), which was performed in accordance with the 1964 Declaration of Helsinki and later amendments. Trial registration at ClinicalTrials.gov: NCT05853263 and NCT01536275.

### Plasma concentrations of HPA hormones and binding proteins

Plasma ACTH concentrations were quantified by double-monoclonal immunoradiometric assay (Brahms Diagnostics, Hennigsdorf, Germany), plasma POMC by enzyme-linked immunosorbent assay (MyBioSource Inc., San Diego, CA, USA) and plasma cortisol (Immunotech, Beckman Coulter) and cortisol-binding globulin (CBG) (Riazen, ZenTech s.a., Liège, Belgium) by competitive radioimmunoassay. Plasma albumin was quantified by the bromocresol-green colorimetric method (Sigma-Aldrich). Plasma-free cortisol was calculated with the Coolens formula adapted for individual albumin and CBG concentrations.

### Statistical analyses

Data are presented as box plots with median, interquartile range (25th–75th percentiles), and the furthest points within 1.5 times the IQR. Differences between groups were analyzed with the use of Mann–Whitney *U*, chi-squared, or Fisher’s exact test, as appropriate. A two-sided *P* value equal to or less than 0.05 was considered statistically significant. All statistical analyses were done with JMP Pro 14 (SAS Institute Inc.).

## Results

**Figure 2:**
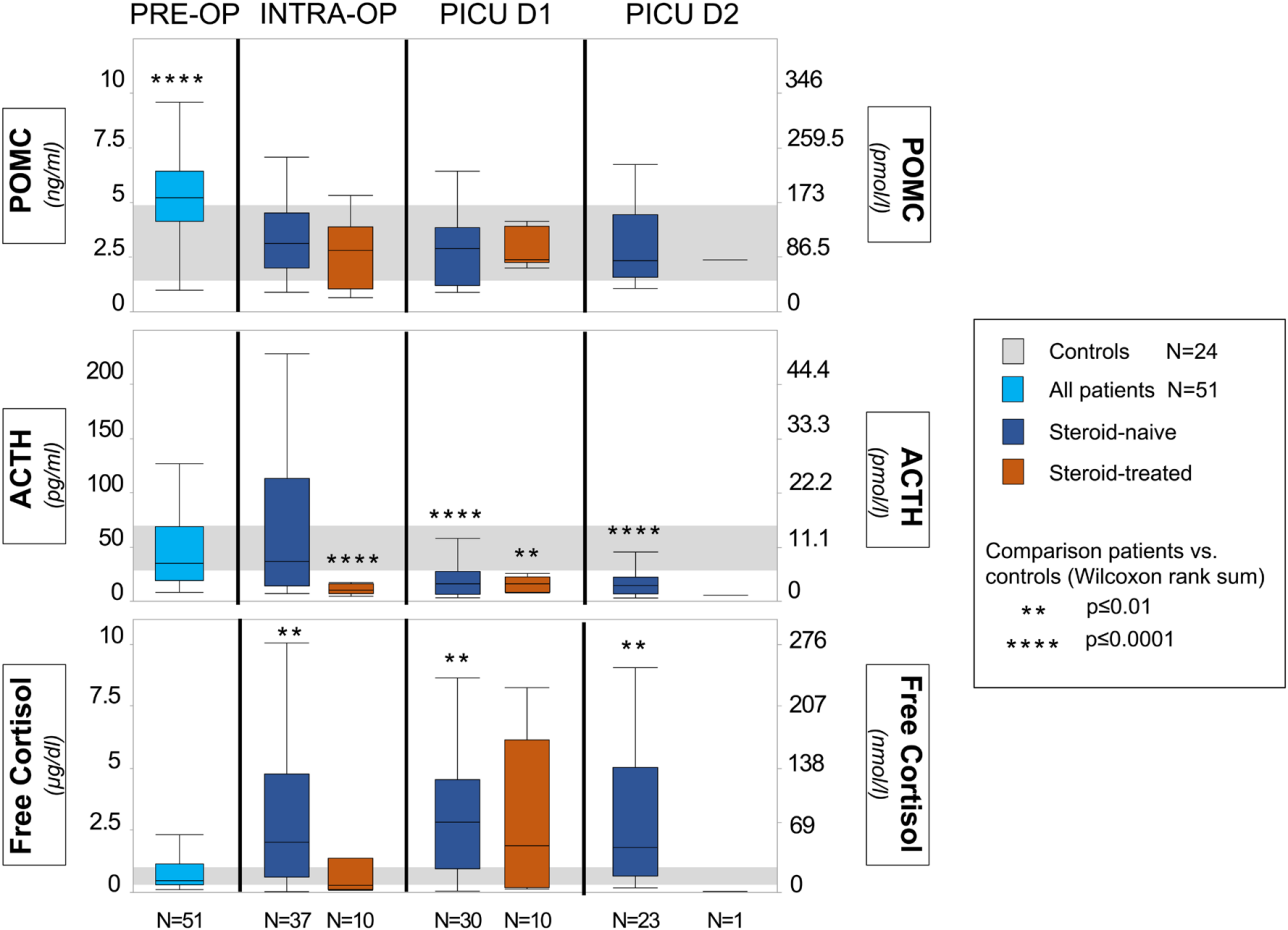
Plasma hormone concentrations pre-, intra-, and post-operatively (PICU days 1 and 2). Box-and-whiskers represent median, interquartile range (IQR), and the furthest points within 1.5 times the IQR of the patients. Gray bars represent IQR of the matched-healthy controls. *P* values above each box-and-whiskers plot indicate significance between the respective patient group and the matched-healthy controls.

In critically ill children who underwent cardiac surgery, plasma POMC concentrations were increased pre-operatively (*P* ≤ 0.0001), as compared with healthy controls, but no longer thereafter (all *P* > 0.05), irrespective of intraoperative glucocorticoid treatment (Fig. 2). Plasma ACTH concentrations were never higher in patients than in healthy children (all *P* > 0.05). While in glucocorticoid-naive patients, plasma ACTH became suppressed only by PICU day 1 (*P* ≤ 0.0001), glucocorticoid-treated patients had already suppressed plasma ACTH intraoperatively (*P* ≤ 0.0001) (Fig. 2). In glucocorticoid-naive patients, plasma-free cortisol was increased from intraoperatively onwards (*P* ≤ 0.01), while in glucocorticoid-treated patients, plasma-free cortisol concentrations were never higher than those of healthy children (all *P* > 0.05) (Fig. 2).

## Discussion

In this study, conducted in critically ill children who underwent cardiac surgery, we first confirmed the well-known hormonal constellation of low plasma ACTH in the face of elevated systemic glucocorticoid availability (i.e. free cortisol) in the early post-operative phase (4, 6, 7). Next, we documented that the plasma concentration of the ACTH precursor POMC was elevated before surgery whereas it decreased to normal levels from the operative phase onwards. Finally, administration of glucocorticoids during surgery was found to accelerate the phenotype of suppressed plasma ACTH with low plasma ACTH already intraoperatively, within minutes to hours after the treatment.

The first important novel finding was the observation of an increase in plasma POMC concentration in the immediate pre-operative phase in children who were scheduled to undergo cardiac surgery with cardiopulmonary bypass. Pre-operatively elevated plasma POMC, not accompanied by increased plasma ACTH, suggests, already in this early phase, the presence of a centrally activated HPA axis with impaired or immature pituitary processing of POMC into ACTH and/or impaired ACTH secretion. Whether this is caused by the physical stress induced by congenital heart disease, or by the stress of hospitalization of young children is unknown.

In the past decade, it has been well documented that plasma ACTH concentrations are suppressed upon ICU admission in both critically ill adults and critically ill children (4, 6). It has also been shown that plasma ACTH is further suppressed by glucocorticoid treatment during the phase of critical illness (3, 4). Here, we have shown that administration of glucocorticoids during surgery, in the pre-ICU phase, results in an acceleration of suppressed plasma ACTH, with low ACTH already observed before the end of surgery. The use of glucocorticoids was at the discretion of the treating physician and not determined by randomization, which is an important limitation of our study. Nevertheless, as low plasma ACTH has been associated with worse outcome in the PICU (4), and as a recent RCT failed to show benefit from prophylactic peri-operative glucocorticoids during cardiac surgery in children (8), the use of glucocorticoids in pediatric cardiac surgery is controversial and questionable (4, 8, 9, 10, 11, 12).

## Conflict of interest

The authors declare that there is no conflict of interest that could be perceived as prejudicing the impartiality of the study reported.

## Funding

This work was supported by the ZonMwhttp://dx.doi.org/10.13039/501100001826 (The Netherlands Organization for Health Researchhttp://dx.doi.org/10.13039/100005622 and Development), ‘effective, efficient, safer use of medicines’ project (project number 836041016), and the Sophia Foundation for Scientific Research (SSWO) (project number S16-08), the European Respiratory Societyhttp://dx.doi.org/10.13039/100008593 (ERS Gold Medal in ARDS to GVdB), the Research Foundation Flanders (FWO) grant G091918N to GVdB, the European Research Councilhttp://dx.doi.org/10.13039/501100000781 Advanced Grant (AdvG-2017-785806 to GVdB) from European Union’s Horizon 2020 research and innovation program, and the Methusalem program of the Flemish Government (METH/14/06 to GVdB, IV and LL via the KU Leuvenhttp://dx.doi.org/10.13039/501100004040).

## Data availability statement

Some or all datasets generated during and/or analyzed during the current study are not publicly available but are available from the corresponding author on reasonable request.
